# Effects of Surface Functional Groups on the Adhesion of SiO_2_ Nanospheres to Bio-Based Materials

**DOI:** 10.3390/nano9101411

**Published:** 2019-10-03

**Authors:** Zuobing Xiao, Jing Xu, Yunwei Niu, Guangyong Zhu, Xingran Kou

**Affiliations:** School of Perfume and Aroma Technology, Shanghai Institute of Technology, No.100 Haiquan Road, Shanghai 201418, China; xzb@sit.edu.cn (Z.X.); 176071219@mail.sit.edu.cn (J.X.); nyw@sit.edu.cn (Y.N.); Zhugyz@sit.edu.cn (G.Z.)

**Keywords:** nanospheres, surface, organically modified silica, leather, isothermal adsorption, interaction

## Abstract

The interactions between nanoparticles and materials must be considered when preparing functional materials. Although researchers have studied the interactions between nanoparticles and inorganic materials, little attention has been paid to those between nanoparticles and bio-based protein materials, like leather. In this study, organically modified silica nanospheres (SiO_2_ nanospheres) loaded with rose fragrance were prepared using (3-aminopropyl) triethoxysilane (APTES), (3-mercaptopropyl) triethoxysilane (MPTES), or 3-(2, 3-epoxypropyloxy) propyl triethoxysilane (GPTES) using the sol-gel method. To study the interactions between the modified SiO_2_ nanospheres and leather, a non-cross-linking adsorption experiment was conducted. According to the Dubinin–Radushkevich isotherm calculation, we found that the adsorption process of leather fiber and organically modified silica nanospheres is physical. The average adhesion energies of APTES-, MPTES-, and GPTES-modified SiO_2_ nanospheres on the leather are 1.34016, 0.97289, and 2.09326 kJ/mol, respectively. The weight gain, adsorption capacity, and average adhesion energy show that the modified SiO_2_ nanospheres can be adsorbed on leather in large quantities. The sensory evaluation confirmed that GPTES-modified SiO_2_ nanospheres endowed the leather with an obvious rose aroma.

## 1. Introduction

The adsorption behavior of nanoparticles can be divided into two categories: adsorbents adsorbing substances, or being adsorbed on the surfaces of materials [1]. Many reports have been published about the former. For example, the adsorption process of Fe_3_O_4_ magnetic nanoparticles to remove Ni (II) is a spontaneous and endothermic process [2]. Maghemite nanoparticles (Fe_2_O_3_) were used to adsorb As(V), and the high adsorption capacity was 50 mg/g [3]. The maximum adsorption capacity of magnetic Ni*_x_*Cu_(1−*x*)_Fe_2_O_4_ (*x*  =  0.1–0.9) nanoparticles for methyl blue was 78.3 mg/g at pH 5 [4]. For the latter category, nanoparticles can be adsorbed on the surfaces of both organic and inorganic materials. Therefore, studying the interaction between nanoparticles and substrate is important for the preparation of functional materials. Modifying the surface of silica particles with hydrophilic –NH_2_ and –SH groups is beneficial to the adsorption of gold nanoparticles, whereas modification with hydrophobic –CH_3_ and –PPh_2_ groups is not conducive to the adsorption of gold nanoparticles [5]. Studying the adsorption behavior of a surfactant onto sandstone rock in the presence of nano-particles was important to improving the performance of chemical stimulations in conventional oil reservoirs [6]. Examination of metal nanoparticles showed that palladium, platinum, and titanium particles strongly chemisorb onto carbon nanotube (CNT) surfaces [7]. The adsorption of gold hydrosol nanoparticles onto the surfaces of polystyrene and poly(2-vinyl pyridine) was found to be irreversible [8]. Although the above researchers studied the interactions between nanoparticles and inorganic materials, little attention has been paid to the interactions between nanoparticles and bio-based protein materials, such as leather.

Nano-silica has a unique three-dimensional silica structure, small particle size, large specific surface area, and good biocompatibility, and is non-toxic, harmless, and pollution-free. Nano-silica also produces a surface interface effect, a small size effect, and a quantum size effect. Therefore, it is widely used in the fields of catalysis, biological imaging, drugs, and gene loading [9,10,11]. Due to its high surface energy, nano-silica easily forms aggregates or secondary aggregates [12,13]. Therefore, the surface of silica nanoparticles must be modified to improve their stability and to disperse them well in various solvents and matrix materials [14,15,16,17].

Until now, organically modified silica has been widely used in the leather industry. The surface of nano-silica is grafted with safranine to dye leather. Compared with common dyeing agents, modified nano-silica has a stronger and more uniform dyeing ability, and considerably enhances the acid, light, and heat resistance of leather [18]. Chitosan-based SiO_2_ nano-capsules were combined with film-forming agents for leather finishing; the leather had good antibacterial properties [19]. Leather finishing agent composed of acrylic resin/nano-silica increased the air permeability of leather by 7.8%, reduced the water absorption by 17.89%, and improved the properties of leather [20]. However, from the previous research reports, researchers only studied the effect of modified nano-silica on leather properties, and research on the interaction mechanisms between modified nano-silica and leather is lacking.

Therefore, in this study, SiO_2_ nanospheres loaded with rose fragrance were prepared using the sol-gel method with 3-aminopropyl triethoxysilane (APTES), 3-mercaptopropyl triethoxysilane (MPTES), or 3-(2, 3-epoxy propylene) propyl triethoxysilane (GPTES) as modifiers. Without any chemical adhesive, an adsorption experiment was conducted between the modified SiO_2_ nanospheres and leather. We explored the leather adsorption differences between the SiO_2_ nanospheres modified with different groups. The effects of different modified nanospheres on leather aroma were investigated. The average adhesion energy was calculated by UV-visible (UV-vis) spectrophotometry and the Dubinin–Radushkevich isotherm adsorption line. We studied the type and strength of the interactions between the different modified nanospheres and leather. The objective of this study was to investigate the effects of surface functional groups on the adhesion of SiO_2_ nanospheres to leather, which will facilitate more modified SiO_2_ nanospheres adsorbing on the leather for endowing the leather with specific properties. This study provides new insights into the interaction between nanospheres and bio-based materials.

## 2. Experiments

### 2.1. Materials and Methods

Tetraethoxy silane (SiC_8_H_20_O_4_; TEOS; analytically pure) was used as the precursor; (3-aminopropyl) triethoxysilane (SiC_9_H_23_O_3_N; APTES; analytically pure), (3-mercaptopropyl) triethoxysilane (SiC_9_H_22_O_3_S; MPTES; analytically pure), and 3-(2, 3-epoxypropyloxy) propyl triethoxysilane (SiC_12_H_26_O_5_; GPTES; analytically pure) were used as the silane coupling agents; hexadecyl trimethyl ammonium bromide [C_16_H_33_ (CH_3_) _3_NBr; CTAB; analytically pure] was used as the surfactant and ammonia hydroxide solution (NH_4_OH; analytically pure) as the catalyst. Anhydrous ethanol (C_2_H_6_O; analytically pure) was used as the solvent. All reagents were purchased from Shanghai Titan Technology Co., Ltd. (Shanghai, China). Full-grain cow leather was provided by Guangzhou Shengyao Environmental Protection Technology Co., Ltd. (Guangzhou, China). Rose fragrance and deionized water were prepared by our laboratory. The rose fragrance was composed of citronella acetate, vanilla acetate, hexol, acetaldehyde, caryophyllene, citral, citronellal, 1% Turkic ketone, eugenol, citronellol formate, phenylethyl formate, citronellol, geranium essential oil, linalool, methyl eugenol, nerol, rose ether, phenyl ethanol, pinene, terpineol, 4-terpinol, and farnesol. The rose fragrance was diluted and mixed with decanoyl- and octanoyl-glycerides in equal proportions. The relative molecular mass and chemical structure of silane precursor and silane coupling agent are shown in Table 1.

### 2.2. Preparation of Organically Modified Silica Rose Fragrance Nanospheres

The organically modified silica rose fragrance nanospheres were prepared according to the improved lavender nanospheres preparation method [21]. The specific operation methods are as follows. First, a mixture of rose fragrance (1.0 g), the silane precursor TEOS (1.0 mL), and silane coupling agents APTES, MPTES, or GPTES (0.5 mL) were fully mixed to form a clear solution. Second, certain concentrations of CTAB, deionized water (28.5 mL), and anhydrous ethanol (14.2 mL) were added in turn, and magnetically stirred for 30 min under at 1500 rpm and 35 °C. A stable microemulsion was formed using an ultrasonic cell breaker for 10 min. An ammonia solution (1.04 mL) was added to catalyze the interfacial hydrolysis condensation reaction between the precursor and the silane coupling agent. The solution was stirred at a speed of 300 rpm for 16 h, and the temperature of the stirring process was 35 °C. Finally, the reaction liquid was cooled to room temperature (25 °C), filtered, and then washed with anhydrous ethanol and deionized water. After freeze-drying at −58 °C for 24 h, organically modified silica rose fragrance nanospheres were obtained.

### 2.3. Characterization of the Morphology and Chemical Structure of Modified SiO_2_ Nanospheres

The SiO_2_ nanospheres prepared with different silane coupling agents were bonded to the conducting resins, which were located on the metal stubs. Before observation, the samples were coated with gold using a gold sputter coater in a high-vacuum evaporator (E-1010 ION SPUTTER, Hitachi, Ltd., Tokyo, Japan). After the pretreatment, a Hitachi S-3400N scanning electron microscope (SEM, Hitachi High-Technologies, Tokyo, Japan) was used to observe the morphology of the nanospheres at an acceleration voltage of 10 kV. The particle size of the modified SiO_2_ nanospheres was analyzed using Nano Measurer 1.2 software [22]. The structure of the modified SiO_2_ nanospheres was characterized using a transmission electron microscope (TEM; Talos F200S, ThermoFisher, Waltham, MA, USA).

The SiO_2_ nanospheres that were modified using different coupling agents and rose fragrance were scanned at full waveband using Fourier transform infrared spectroscopy (Bruker, Billerica, MA, USA) in the range of 500 to 4000 cm^−1^. The obtained infrared spectrograms were used to investigate the chemical structures of the modified SiO_2_ nanospheres. X-ray powder diffraction (XRD; X’Pert PRO MPD, Nalytical, Netherlands) was used to characterize the crystal types of modified SiO_2_ nanospheres.

### 2.4. Application of Modified SiO_2_ Nanospheres on Leather

The leather was cut into small pieces (10 × 10 cm) with scissors. The solution was prepared using ammonia, anhydrous ethanol, and deionized water in a weight ratio of 1:2:17. After the solution was mixed evenly, dry gauze was dipped into the solution. The gauze with the solution was used to gently wipe the leather surface to remove the dust and dirt, and to clean the leather surface. After, the wet leather was placed in a dry and ventilated place, and then was placed into a pp bag after the leather surface was completely dry.

The modified SiO_2_ nanospheres were added to 100 mL deionized water and then vibrated using a rotary shaking table (ZHWY-304, Zhicheng analytical instrument manufacturing co. LTD, Shanghai, China) at 110 rpm and 25 °C for 30 min to form a dispersed solution. The leather with a clean surface was immersed in the solution, and the duration of adsorption was 4 h. Then, the wet leather was placed in a dry and ventilated place at 25 °C for 24 h to completely dry the leather. Finally, the nano-encapsulated aromatic leather was obtained.

### 2.5. Characterization of Aromatic Leather

The leather that was soaked in the solution of SiO_2_ nanospheres modified with different coupling agents was bonded to the conductive resin on the metal post. Before observation, the samples were plated with a gold sputter coating machine in a high vacuum evaporator (E-1010 ION SPUTTER, Hitachi, Ltd., Tokyo, Japan). After pretreatment, the morphology of the aromatic leather was observed using a Hitachi S-3400N scanning electron microscope (Hitachi High Technology, Tokyo, Japan) under a 10 kV accelerated voltage.

The dried weights of the leather before and after soaking were accurately weighed using electronic scales (AL204, Mettler Toledo, Shanghai, China) to calculate the weight gain. A thermogravimetric analyzer (Q5000IR; TA Instruments, New Castle, DE, USA) was used to explore the thermal stability of the modified SiO_2_ nanospheres. The sample injection volume was 3 to 5 mg. The whole process was conducted with the protection of N_2_. The test temperature programming was controlled ranging from 30 to 600 °C, and the heating rate was 10 °C·min^−1^. On the basis of the above thermogravimetric analysis results, the capacity of leather to adsorb modified SiO_2_ nanospheres was calculated [23].

We invited 10 evaluators (5 men, 5 women; 20–30 years old) who had received sensory evaluation training to evaluate the aroma quality of the leather. The aroma intensity of the aromatic leather was scored on a scale ranging from 0 to 9, with 0 indicating the weakest rose aroma intensity and 9 indicating the strongest rose aroma intensity.

### 2.6. Interaction Between Modified SiO_2_ Nanospheres and Leather

The SiO_2_ nanospheres modified using different coupling agents were dispersed into a deionized aqueous solution, and the linear Beer–Lambert standard curve was determined using UV-vis spectrophotometer (Alpha-1860, Shanghai Element, Shanghai, China). We conducted an experiment using leather and modified SiO_2_ nanospheres without cross-linking agent adsorption. Simultaneously, the concentration of the modified SiO_2_ nanospheres, which were adsorbed by leather in the initial solution, was analyzed using a UV-vis spectrophotometer at a wavelength of 200 nm. After adsorption equilibrium, the concentration of the modified SiO_2_ nanospheres in the residual solution was measured by UV-vis spectrophotometry at a wavelength of 200 nm. All the above experiments were performed three times. The concentration analysis was based on the linear Beer–Lambert standard curve established previously. The adsorption capacity of modified SiO_2_ nanospheres on leather was determined as follows [24]:

q_e_ = V(C_0_ − C_e_)/M (1)

where q_e_ (mg/g) is the capacity of leather to adsorb modified SiO_2_ nanospheres, C_0_ (mg/L) is the initial concentration of modified SiO_2_ nanospheres in dispersion, C_e_ (mg/L) is the concentration of modified SiO_2_ nanospheres in the residual solution, V (L) is the volume of dispersion, and M (g) is the quality of the leather used for adsorption. To determine the adsorption capacity of SiO_2_ nanospheres on the leather, the equilibrium experimental data were analyzed with Langmuir, Freundlich, and Dubinin–Radushkevich isotherm models. The linearized equation of the Langmuir isotherm model can be expressed as:

C_e_/q_e_ = (1/K_L_Q_0_) + C_e_/Q_0_, (2)

where C_e_ is the concentration of modified SiO_2_ nanospheres in residual solution (mg/L), q_e_ (mg/g) is the capacity of leather to adsorb modified SiO_2_ nanospheres, Q_0_ is the maximum adsorption capacity of the modified SiO_2_ nanospheres (mg/g), and K_L_ is the adsorption equilibrium constant (L/mg).

The Freundlich isotherm is an empirical equation based on the adsorption on a heterogeneous surface and assumes that the adsorption occurs at sites with different adsorption energies. The equation is commonly expressed as:

ln(q_e_) = ln(K_F_) + (1/n)ln(C_e_), (3)

where K_F_ (mg/g) and n are the Freundlich constants characteristics.

The expression of the Dubinin–Radushkevich isotherm adsorption model is as follows [2,24]:

ln(q_e_) = ln(q_m_) – K_D_ε^2^″ (4)

ε = RTln [1 + (1/C_e_)]′ (5)

where q_m_ is the maximum adsorption capacity of the modified SiO_2_ nanospheres (mg/g), R is the ideal gas constant (8.314 kJ·mol^−1^K^−1^), T is the adsorption temperature (K), and K_D_ is the Dubinin–Radushkevich isotherm adsorption constant (mol^2^·kJ^−2^). According to K_D_, the average adhesion energy of the adsorption process can be calculated.

According to the fitting parameters of the Dubinin–Radushkevich equation, the average adhesion energy E (kJ/mol) of leather adsorption modified SiO_2_ nanospheres was calculated as follows:

(6) $$E=\frac{1}{\sqrt{2K_{D}}},$$

where K_D_ is the Dubinin–Radushkevich isotherm adsorption constant (mol^2^ kJ^−2^).

## 3. Results and Discussion

### 3.1. Preparation of Modified SiO_2_ Nanospheres Using the Sol-Gel Method

**Figure 1:**
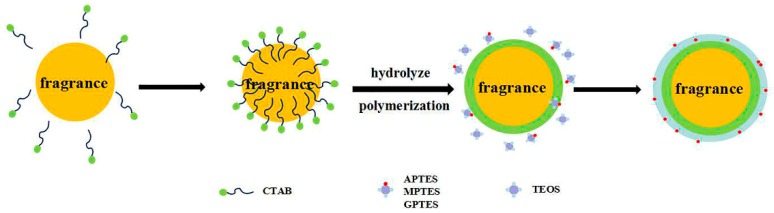
Schematic diagram of the reaction steps of organically modified silica (ORMOSIL) rose fragrance nanospheres.

The sol-gel method was used to prepare rose fragrance nanospheres coated with organically modified silica. Hydrolysis and polycondensation of the precursor and coupling agent at the two-phase interface of the microemulsion were completed, and the reaction mechanism is shown in Figure 1. Rose fragrance, organosilane precursor (TEOS), silane coupling agents (APTES, MPTES, and GPTES), CTAB, deionized water, and anhydrous ethanol were mixed and emulsified at 1500 rpm to form stable oil-in-water (O-W) microemulsion. When the ammonia hydroxide solution was added to the microemulsion system, the silane precursor and the coupling agent reacted via hydrolysis and condensation to form a negatively charged organically modified silica oligomer and silicone alcohol. The negatively charged products were adsorbed by positively charged CTAB on the surface of oil droplets. With the reaction progression, the precursor and the coupling agent continuously diffused to the water/oil interface and formed a complete organically modified silica wall material on the surface of the rose fragrance.

### 3.2. Morphology Analysis of Modified SiO_2_ Nanospheres

**Figure 2:**
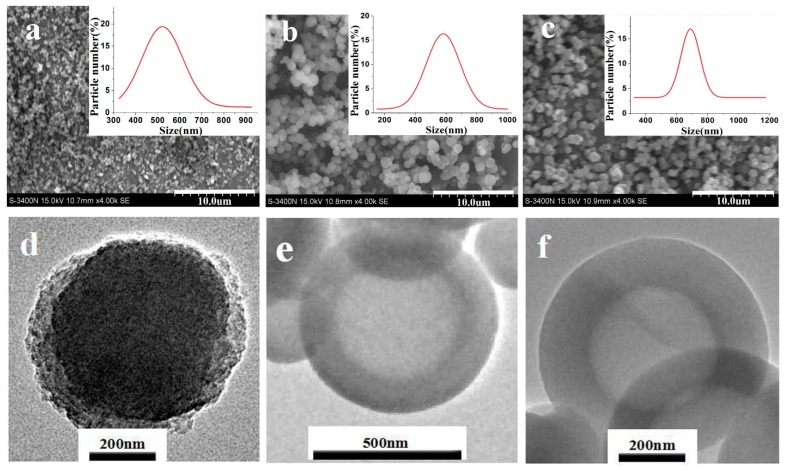
SEM images of (**a**) APTES-modified, (**b**) MPTES-modified, and (**c**) GPTES-modified SiO_2_ nanospheres; TEM images of (**d**) APTES-modified, (**e**) MPTES-modified, and (**f**) GPTES-modified SiO_2_ nanospheres; the insets in the SEM images are the particle size distributions of SiO_2_ nanospheres modified by different functional groups.

Figure 2 shows the SEM, TEM, and particle size distribution of modified SiO_2_ nanospheres. The particle size distribution graph shows that the average particle size of the modified SiO_2_ nanospheres was mainly distributed in the range of 600 to 700 nm (± 50 nm). The average particle size of SiO_2_ nanospheres modified by APTES, MPTES, and GPTES was 551 ± 50, 581 ± 50, and 688 ± 50 nm, respectively. Their particle size distribution graphs show a normal distribution, indicating that the size of the modified SiO_2_ nanospheres was uniform. By comparing Figure 2a–c, the SiO_2_ nanospheres modified by MPTES and GPTES have good dispersibility. The TEM results in Figure 2e,f also demonstrate the core-shell structure. The shell thicknesses of MPTES nanospheres and GPTES nanospheres are 108.21 and 154.34 nm, respectively. The SiO_2_ nanospheres modified by APTES were the most agglomerated, potentially due to the introduction of –NH_2_, which endowed the strong silicon interaction with –OH among the nanospheres.

### 3.3. Fourier Infrared Spectrum and XRD Study of Modified SiO_2_ Nanospheres

**Figure 3:**
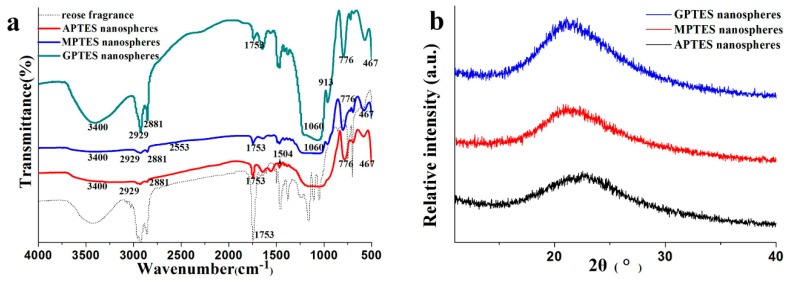
(**a**) Fourier transform infrared (FTIR) spectroscopy of modified SiO_2_ nanospheres; (**b**) X-ray powder diffraction (XRD) spectroscopy of modified SiO_2_ nanospheres.

The Fourier transform infrared (FTIR) results of the modified SiO_2_ nanospheres are shown in Figure 3a. We analyzed the infrared spectra of the SiO_2_ nanospheres that were modified by different coupling agents and rose fragrance. The wavenumber near 3400 cm^−1^ is the stretching vibration absorption peak of Si–OH, 1060 cm^−1^ and 776 cm^−1^ are the antisymmetric stretching vibration peak of Si–O–Si and the symmetrical stretching vibration peak of Si–O, respectively. The bending vibration peak of Si–O is located at 467 cm^−1^. These spectral bands are the functional groups of silica, and they indicate that the condensation and polymerization between TEOS and APTES, MPTES, and GPTES were successful. From the infrared spectra of the modified SiO_2_ nanospheres, we observed that in addition to the vibration peak of Si–O in the structure, the vibration absorption peaks at 2929 cm^−1^ and 2881 cm^−1^ belonged to the antisymmetric and symmetric telescopic vibration of C–H in –CH_3_ and –CH_2_, respectively. The characteristic peaks of the three different types of silane coupling agents also appeared. For example, the characteristic absorption peaks of –NH_2_ [25], –SH, and epoxy groups occur at 1504, 2553, and 913 cm^−1^, respectively. These results showed that different functional groups had been successfully grafted onto silica nano-fragrance using APTES, MPTES, and GPTES. The characteristic absorption peak of rose fragrance was observed near 1753 cm^−1^, indicating that rose fragrance was encapsulated in nanospheres with modified silica as a shell. From Figure 3b, the XRD of nanospheres showed a wide and dispersive peak at 2θ ≈ 22.5° [26], indicating that the modified SiO_2_ nanospheres existed in amorphous form.

### 3.4. Morphology Analysis of Modified SiO_2_ Nanospheres Leather

**Figure 4:**
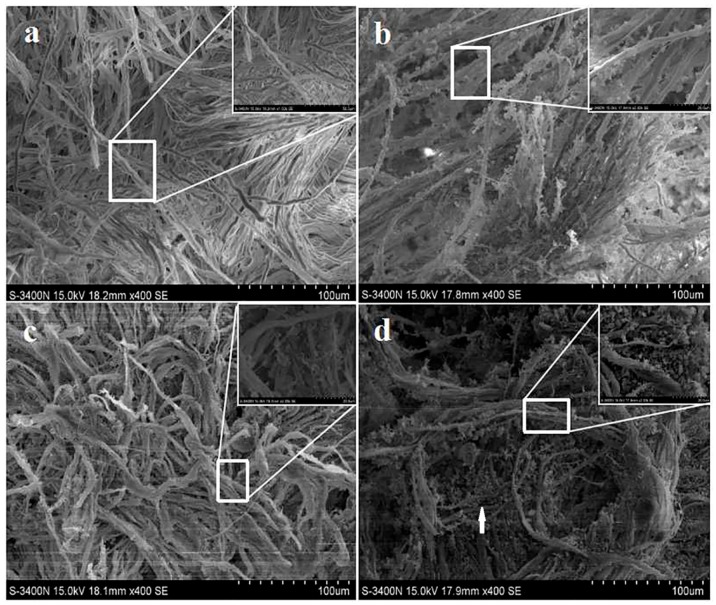
SEM images of modified SiO_2_ nanospheres leather without a cross-linking agent: (**a**) blank leather, (**b**) APTES-modified, (**c**) MPTES-modified, and (**d**) GPTES-modified SiO_2_ nanospheres leather.

Figure 4a shows that the untreated leather had many collagen fibers with a smooth surface and uniform thickness. These observations are similar to those reported in the literature [27]. Many cracks were observed between the leather collagen fibers [28]. The leather treated with the modified SiO_2_ nanospheres exhibited good adhesion to the leather surface, as shown in Figure 4b–d. The modified SiO_2_ nanospheres can be absorbed into the collagen fibers. Compared with the leather treated with the other two modified SiO_2_ nanospheres, more nanospheres were observed on the sample surface treated with epoxy-group-modified nanospheres. This may be due to the epoxy group reacting with the active groups such as the amino group, carboxyl group, and amide group on the collagen fiber [29]. This interaction may form hydrogen bonds [30], van der Waals forces, and electrostatically interact to promote more modified SiO_2_ nanospheres to bind to the collagen fiber.

### 3.5. Thermogravimetric Analysis and Sensory Evaluation

**Figure 5:**
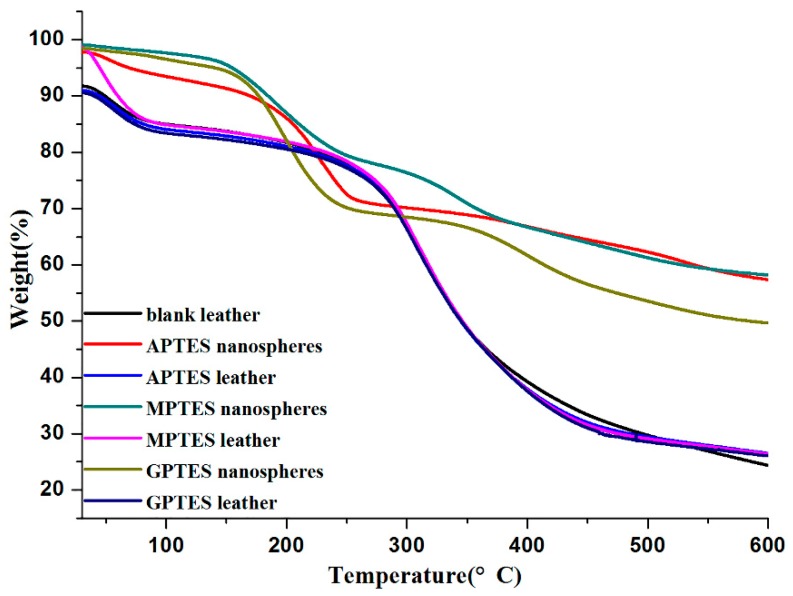
Thermogravimetric analysis of modified SiO_2_ nanospheres and non-crosslinker-modified SiO_2_ nanosphere leather.

The thermogravimetric (TG) analysis of modified SiO_2_ nanospheres is shown in Figure 5. The weight of modified SiO_2_ nanospheres decreased with increasing temperature. The thermal degradation process of modified SiO_2_ nanospheres can be divided into three steps. In the first stage, the thermal decomposition of 30–200 °C was due to the weight loss of water and fragrance. In the second stage, the weight loss of 200–350 °C can be explained by the rapid release of the fragrance, which was the core material, and the decomposition of the wall material. In the third stage, the weight loss of 350–600 °C is the decomposition of the wall material. The TG curve also showed that 50% of the modified SiO_2_ nanospheres still had mass residues even at 600 °C, which proved that the organically modified silica had good thermal stability as a wall material.

The thermogravimetric analysis curve showed that the weight loss rate of blank leather without adsorbed modified SiO_2_ nanospheres was 67.45% after heating to 600 °C. The weight loss rates of leather adsorption of the APTES, MPTES, GPTES modified SiO_2_ nanospheres were 64.58%, 65.59%, and 64.63% at 30–600 °C, respectively. This was due to the residue of modified SiO_2_ nanospheres, which confirmed that leather adsorbed modified SiO_2_ nanospheres.

The dried weights of the leather before and after soaking were accurately weighed using electronic scales, and all the dried weights of leather increased. Among the leather with increased weight, that which was soaked in GPTES-modified SiO_2_ nanospheres dispersion solution increased the most. The capacity of leather to adsorb modified SiO_2_ nanospheres were calculated from the thermogravimetric curve, and the results are shown in Table 2. The adsorption capacity calculation showed that the modified SiO_2_ nanospheres were adsorbed on leather in the order of GPTES, APTES, and MPTES. The weight gain and the adsorption capacity together indicate that the capacity of leather adsorb GPTES-modified SiO_2_ nanospheres was the strongest, and the ability to adsorb MPTES-modified SiO_2_ nanospheres was relatively weak. This may be due to the different functional groups on the surface of SiO_2_ nanospheres. These functional groups had different reactive activities, and they can form hydrogen bonds or other intermolecular interactions with amino groups, carboxyl groups, and other functional groups on leather collagen fibers. Due to the interaction, the modified SiO_2_ nanospheres can be adsorbed on leather without a cross-linking agent. In addition, the epoxy group of SiO_2_ nanospheres modified by GPTES had relatively strong activity, so more easily reacted with the active functional groups on collagen fibers to form strong interactions between the modified SiO_2_ nanospheres and the leather. This strong interaction may promote the leather adsorbing more GPTES-modified SiO_2_ nanospheres.

We invited 10 evaluators to evaluate the aroma quality of leather soaked in SiO_2_ nanospheres that were modified with different coupling agents. The results are shown in Table 3. Through statistical calculation, the leather treatment with SiO_2_ nanospheres modified by GPTES received the highest score. This may be due to the leather having the strongest ability to adsorb GPTES-modified SiO_2_ nanospheres.

### 3.6. Adsorption Isotherm

**Figure 6:**
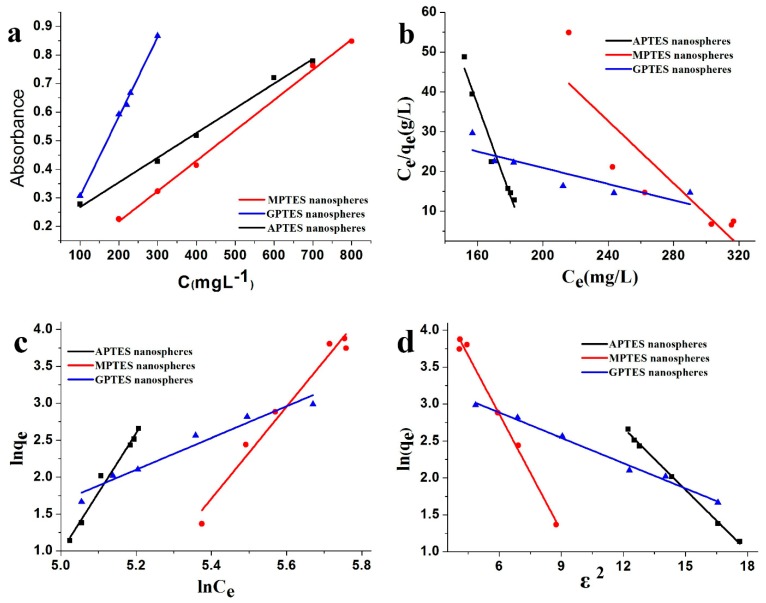
(**a**) Beer–Lambert standard curve of modified SiO_2_ nanospheres; (**b**) Langmuir adsorption isotherm and (**c**) Freundlich adsorption isotherm, and (**d**) Dubinin–Radushkevich adsorption isotherm of leather adsorption of modified SiO_2_ nanospheres.

The Beer–Lambert standard curve of modified SiO_2_ nanospheres was determined using the UV-vis absorbance method as shown in Figure 6a. The coefficient of determination (*R*^2^) > 0.99 of the standard curve showed that the standard curve had an excellent linear relationship. Figure 6d shows that the Dubinin–Radushkevich isotherm adsorption model was suitable for application to leather adsorption of modified SiO_2_ nanospheres. The *R*^2^ > 0.99 indicated that the fitting curve had good linearity. The Langmuir [2,24,31] and Freundlich [2] isotherm adsorption models of leather adsorption modified SiO_2_ nanospheres are provided in Figure 6b,c. The *R*^2^ was less than 0.99, and the fitting curve did not have a good linear relationship. The results show that the leather adsorption modified SiO_2_ nanospheres do not conform to the Langmuir and Freundlich isotherm adsorption models. The results of K_D_, which were obtained from the adsorption isotherms, are shown in Table 4. The average adhesion energies of the SiO_2_ nanospheres with modified by APTES, MPTES, and GPTES are 1.34016, 0.97289, and 2.09326 kJ/mol, respectively. Since the values average adhesion energy E were all less than 8 kJ/mol, the adsorption process is physical [32]. In other words, hydrogen bonds or van der Waals forces between modified SiO_2_ nanospheres and leather collagen fibers may play roles to make the nanospheres adsorbed on the surface of the leather. By comparing the average adhesion energies E, we found that the average adhesion energy of leather that adsorbed the GPTES-modified SiO_2_ nanospheres was the largest. The second was the APTES-modified SiO_2_ nanospheres that were adsorbed on the leather, and the average adhesion energy of the MPTES-modified SiO_2_ nanospheres was the lowest. The result showed that leather had a strong adsorption capacity for GPTES-modified SiO_2_ nanospheres. This result is consistent with the leather weight gain of modified SiO_2_ nanospheres and the capacity of leather to adsorb modified SiO_2_ nanospheres.

## 4. Conclusions

In this study, to investigate the interaction between modified SiO_2_ nanospheres and leather, three kinds of modified SiO_2_ nanospheres loaded with rose fragrance were prepared with the sol-gel method using APTES, MPTES, or GPTES. The weight gain, adsorption capacity, and average adhesion energy calculation showed that modified SiO_2_ nanospheres can be physically adsorbed on leather in the order of GPTES, APTES, MPTES. The sensory evaluation confirmed that GPTES-modified SiO_2_ nanospheres endowed the leather with an obvious rose aroma. These results indicate that the surface functional groups play roles in the adhesive capacity of SiO_2_ nanospheres on the leather surface. Among the three tested active groups, the epoxy group endowed SiO_2_ nanospheres with the best adhesive capacity. This paper not only outlined a promising method for fragrance adhesion to leather surfaces without cross-linking agents but also provided insight into the interaction between nanospheres and bio-based materials.

## Figures and Tables

**Table 1:** Relative molecular mass and chemical structural formula of silane coupling agent.

Agent	Chemical Construction	Relative Molecular Mass
TEOS	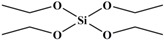	208
APTES	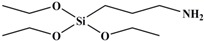	221
MPTES	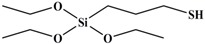	238
GPTES	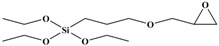	278

**Table 2:** Weight gain and adsorption capacity of SiO_2_ nanospheres leather without a cross-linking agent.

Silane Coupling Agent	Mass Before Adsorption (g)	Mass After Adsorption (g)	Δ (g)	Adsorption Capacity (mg/g)
APTES	2.6846	2.8165	0.1319	26.5760
MPTES	2.8559	2.9800	0.1241	15.3520
GPTES	2.9367	3.1000	0.1633	34.2577

**Table 3:** Sensory evaluation of leather absorbing the modified SiO_2_ nanospheres.

Evaluator	APTES-Modified SiO_2_ Nanospheres Leather	MPTES-Modified SiO_2_ Nanospheres Leather	GPTES-Modified SiO_2_ Nanospheres Leather
1	7.0	5.0	9.0
2	6.0	5.0	9.0
3	8.0	6.0	8.0
4	9.0	7.0	9.0
5	8.0	4.0	9.0
6	9.0	6.0	8.0
7	8.0	7.0	9.0
8	7.0	6.0	8.0
9	8.0	7.0	9.0
10	8.0	6.0	9.0
Average score	7.8	5.9	8.7

**Table 4:** Correlation fitting coefficient of SiO_2_ nanospheres modified by leather adsorption.

Nanospheres	K_D_ (mol^2^kJ^−2^)	E (kJ/mol)	Beer–Lambert Standard Curve *R^2^*	Dubinin–Radushkevich Adsorption Isotherm *R^2^*
APTES	0.27839	1.34016	0.99340	0.99710
MPTES	0.52825	0.97289	0.99757	0.99023
GPTES	0.11411	2.09326	0.99340	0.99083

## References

[1] Wu Z., Ye X. (2010). Adsorption of nanoparticles on solid substrate matrix. Mater. Guide.

[2] Gautam R.K., Gautam P.K., Banerjee S., Soni S., Singh S.K., Chattopadhyaya M.C. (2015). Removal of Ni(II) by magnetic nanoparticles. J. Mol. Liq..

[3] Tuutijarvi T., Lu J., Sillanpaa M., Chen G. (2009). As(V) adsorption on maghemite nanoparticles. J. Hazard. Mater..

[4] Pan S., Huang W., Yu Q., Liu X., Liu Y., Liu R. (2019). A rapid combustion process for the preparation of NixCu(1−x)Fe2O4 nanoparticles and their adsorption characteristics of methyl blue. Appl. Phys. A.

[5] Westcott S.L., Oldenburg S.J., Lee T.R., Halas N.J. (1998). Formation and Adsorption of Clusters of Gold Nanoparticles onto Functionalized Silica Nanoparticle Surfaces. Langmuir ACS J. Surf. Colloids.

[6] Ahmadi M.A., Shadizadeh S.R. (2013). Induced effect of adding nano silica on adsorption of a natural surfactant onto sandstone rock: Experimental and theoretical study. J. Pet. Sci. Eng..

[7] Park N., Sung D., Lim S., Moon S., Hong S. (2009). Realistic adsorption geometries and binding affinities of metal nanoparticles onto the surface of carbon nanotubes. Appl. Phys. Lett..

[8] Sukhov V.M., Dement’eva O.V., Kartseva M.E., Rudoy V.M., Ogarev V.A. (2004). Metal Nanoparticles on Polymer Surfaces: 3. Adsorption Kinetics of Gold Hydrosol Particles on Polystyrene and Poly(2-vinylpyridine). Colloid J..

[9] Cao S., Jin X., Yuan X., Wu W., Hu J., Sheng W. (2010). A facile method for the preparation of monodisperse hollow silica spheres with controlled shell thickness. J. Polym. Sci. Part A Polym. Chem..

[10] Wang X., Wang P., Jiang Y., Su Q., Zheng J. (2014). Facile surface modification of silica nanoparticles with a combination of noncovalent and covalent methods for composites application. Compos. Sci. Technol..

[11] Si Y., Guo Z. (2015). Superwetting Materials of Oil–Water Emulsion Separation. Chem. Lett..

[12] Park J.Y., Back S.H., Chang S.J., Lee S.J., Lee K.G., Park T.J. (2015). Dopamine-assisted synthesis of carbon-coated silica for PCR enhancement. ACS Appl. Mater. Interfaces.

[13] Shen Z., Zhu Y., Wu L., You B., Zi J. (2010). Fabrication of robust crystal balls from the electrospray of soft polymer spheres/silica dispersion. Langmuir ACS J. Surf. Colloids.

[14] Lin Y., Zhang A., Sun J., Wang L. (2013). Properties of Natural Rubber Vulcanizates/Nanosilica Composites Prepared Based on the Method of In-situ Generation and Coagulation. J. Macromol. Sci. Part B.

[15] Yin J., Deng T., Zhang G. (2012). Preparation and size control of highly monodisperse vinyl functionalized silica spheres. Appl. Surf. Sci..

[16] Peng Z., Li Q., Li H., Hu Y. (2017). Polyethylene-Modified Nano Silica and Its Fine Dispersion in Polyethylene. Ind. Eng. Chem. Res..

[17] Singh R., Bapat R., Qin L., Feng H., Polshettiwar V. (2016). Atomic Layer Deposited (ALD) TiO2 on Fibrous Nano-Silica (KCC-1) for Photocatalysis: Nanoparticle Formation and Size Quantization Effect. ACS Catal..

[18] Ramalingam S., Sreeram K.J., Raghava Rao J., Unni Nair B. (2016). Organic Nanocolorants: Self-Fixed, Optothermal Resistive, Silica-Supported Dyes for Sustainable Dyeing of Leather. ACS Sustain. Chem. Eng..

[19] Fan Q., Ma J., Xu Q., Wang J., Ma Y. (2018). Facile Synthesis of Chitosan-Coated Silica Nanocapsules via Interfacial Condensation Approach for Sustained Release of Vanillin. Ind. Eng. Chem. Res..

[20] Ma J., Hu J., Yang Z. (2007). Preparation of Acrylic Resin/Nano-SiO2for Leather Finishing Agent. Mater. Manuf. Process..

[21] Xiao Z., Liu M., Niu Y., Zhu G., Deng J., Liu S. (2019). Lavender fragrance sol-gel encapsulated in ORMOSIL nanospheres. Flavour Fragr. J..

[22] Zhu Y., Liang S., Chen K., Gao X., Chang P., Tian C., Wang J., Huang Y. (2015). Preparation and properties of nanoencapsulated n-octadecane phase change material with organosilica shell for thermal energy storage. Energy Convers. Manag..

[23] Wang M., Fu H., She Y., Xiao Z., Zhu G., Hu J. (2015). Adsorption capacity, kinetics, and thermodynamics of chitosan nanoparticles onto cotton fabrics without any chemical binders. Polym. Compos..

[24] Chien-To Hesieh H.T. (2000). Langmuir and Dubinin–Radushkevich analyses on equilibrium adsorption of activated carbon fabrics in aqueous solutions. J. Chem. Technol. Biotechnol..

[25] Visa M., Chelaru A.-M. (2014). Hydrothermally modified fly ash for heavy metals and dyes removal in advanced wastewater treatment. Appl. Surf. Sci..

[26] Yang R., Hu T.D., Liu T., Xiang H. (1998). Characterization of CuO-BaO/SiO_2_ Catalysts structure. Acta Phys. -Chim. Sin..

[27] Fathima N.N., Dhathathreyan A., Ramasami T. (2002). Mercury Intrusion Porosimetry, Nitrogen Adsorption, and Scanning Electron Microscopy Analysis of Pores in Skin. Biomacromolecules.

[28] Wang S., Ye L., Liu Y., Zhu Z., Liu J., Xiao Z., Shen Y., Jiang L. (2019). Fibrous pore structure of silk fabric, cattle leather and wallpaper base paper and their adsorption properties. Sci. Sin. Chim..

[29] Gong J., Dan W., Dan N. (2018). Study on the modification of decellularized porcine dermal matrix with quercetin. Leather Sci. Eng..

[30] Hui P., Zhijun Z., Juxian Z., Zhishen W., Hongxin D. (2004). Preparation and application of a new nanocomposite tanning agent MPNS/SMA. China Leather.

[31] Liu D., Hao L., Fang K. (2014). Adsorption of cationic copolymer nanospheres onto cotton fibers investigated by a facile nephelometry. Colloids Surf. A Physicochem. Eng. Asp..

[32] Onyango M.S., Kojima Y., Aoyi O., Bernardo E.C., Matsuda H. (2004). Adsorption equilibrium modeling and solution chemistry dependence of fluoride removal from water by trivalent-cation-exchanged zeolite F-9. J. Colloid Interface Sci..

